# Minimizing System Entropy: A Dual-Phase Optimization Approach for EV Charging Scheduling

**DOI:** 10.3390/e27030303

**Published:** 2025-03-14

**Authors:** Wenpeng Yuan, Lin Guan

**Affiliations:** School of Electric Power Engineering, South China University of Technology, Guangzhou 510640, China; lguan@scut.edu.cn

**Keywords:** minimizing system entropy, electric vehicle charging, distribution networks, particle swarm optimization, Q-learning, rural power systems

## Abstract

To address the electric vehicle (EV) charging scheduling problem in rural distribution networks, this study proposes a novel two-phase optimization strategy that combines particle swarm optimization (PSO) and Q-learning for global optimization and real-time adaptation. In the first stage, PSO is used to generate an initial charging plan that minimizes voltage deviations and line overloads while maximizing user satisfaction. In the second phase, a Q-learning approach dynamically adjusts the plan based on real-time grid conditions and feedback. The strategy reduces the system’s entropy by minimizing the uncertainty and disorder in power distribution caused by variable EV charging loads. Experimental results on a 33-bus distribution system under baseline and high-load scenarios demonstrate significant improvements over conventional dispatch methods, with voltage deviation reduced from 5.8% to 1.9%, maximum load factor reduced from 95% to 82%, and average customer satisfaction increased from 75% to 88%. While the computation time increases compared to standalone PSO (66 min vs. 34 min), the enhanced grid stability and customer satisfaction justify the trade-off. By effectively minimizing system entropy and balancing grid reliability with user convenience, the proposed two-phase strategy offers a practical and robust solution for integrating EVs into rural power systems.

## 1. Introduction

The rapid proliferation of electric vehicles (EVs) has introduced both opportunities and challenges to modern power systems. While EVs promise reduced carbon emissions and enhanced energy flexibility, their uncoordinated charging behaviors impose a significant strain on distribution networks, particularly in rural and urban–rural transition zones. These regions often face infrastructure limitations, such as weaker grid resilience and lower redundancy, making them more vulnerable to voltage instability, line overloads, and load fluctuations during peak EV charging periods [1,2]. Addressing these challenges requires advanced scheduling strategies that harmonize grid stability with user satisfaction—a dual objective complicated by the inherent uncertainty of EV charging patterns and dynamic grid conditions.

Existing research on EV charging optimization spans a spectrum of methodologies. **Traditional optimization approaches**, such as mixed-integer linear programming (MILP) [3,4] and genetic algorithms (GAs) [5,6], have demonstrated efficacy in minimizing grid impacts under static conditions. For instance, Li et al. [3] proposed a MILP-based framework to reduce voltage deviations in urban grids, yet their model assumes deterministic charging demands, limiting applicability to rural networks with higher variability. **Reinforcement learning (RL)** methods, including Q-learning and deep Q-networks (DQNs), offer dynamic adaptability by learning optimal policies through real-time interactions [7,8]. Zhang et al. [9] applied DQNs to urban EV scheduling, achieving a 12% reduction in peak load; however, their work overlooks the unique voltage constraints of rural grids. **Hybrid strategies** combining global and real-time optimization have recently emerged. For example, Kumar et al. [10] integrated particle swarm optimization (PSO) with RL for microgrid energy management, reporting improved voltage stability. Nonetheless, their focus on urban microgrids leaves rural distribution networks—where line resistances and load profiles differ markedly—underserved. A critical gap persists: few studies holistically address the interplay between global scheduling, real-time adaptability, and the entropy-like disorder introduced by stochastic EV loads in rural systems.

Entropy, a measure of system disorder, provides a compelling lens to quantify the destabilizing effects of EV charging. In power systems, voltage fluctuations and load imbalances can be analogized to entropy increases, where uncontrolled charging amplifies grid unpredictability [11,12]. Recent work by Chen et al. [11] conceptualized entropy reduction in smart grids via renewable integration but did not extend this to EV scheduling. Similarly, Torkan et al. [5] employed entropy metrics for load balancing in urban networks, yet their static framework lacks real-time responsiveness. These gaps underscore the need for an entropy-guided optimization strategy that bridges theoretical rigor with practical adaptability in rural contexts.

In this paper, we propose a novel dual-phase optimization strategy specifically designed for rural distribution networks. Our approach combines particle swarm optimization (PSO) for global charging schedule generation with Q-learning for real-time adaptive adjustments. This integration allows for both system-wide optimization and dynamic response to changing grid conditions. The key innovation of our work is framing the EV charging problem through the lens of system entropy, where we quantify and minimize the disorder in power distribution caused by stochastic EV loads. Our strategy simultaneously optimizes for three critical objectives: minimizing voltage deviations, preventing line overloads, and maximizing user satisfaction. Our contributions are threefold:

- **A novel entropy-driven objective function** that explicitly links voltage deviations, line overloads, and user dissatisfaction to system disorder, providing a unified metric for optimization.
- **A hybrid PSO-Q-learning architecture** that combines global scheduling (via PSO) with real-time adjustments (via Q-learning), tailored to rural grids’ high-resistance lines and variable loads.
- **Comprehensive validation** under both baseline and high-stress scenarios, demonstrating significant improvements in voltage stability (65.5% reduction in deviation), load balancing, and user satisfaction compared to state-of-the-art methods.

By embedding entropy minimization into the optimization core, this work advances the integration of EVs into rural power systems while offering a replicable framework for entropy-aware grid management.

## 2. Related Work

### 2.1. Electric Vehicle Charging Scheduling

Integrating EVs into existing power grids presents significant challenges, particularly in managing charging loads and preventing grid overload. Several studies have focused on optimizing EV charging schedules to alleviate these issues. For instance, refs. [13,14] proposed an optimization framework for residential EV charging, considering factors such as charging power, time slots, and EV capacity, and employing multi-objective optimization to minimize the impact on the distribution network. However, these methods often struggle with scalability and real-time adaptability, especially under varying grid conditions.

Similarly, refs. [3,4] utilized MILP to optimize EV charging schedules, aiming to reduce voltage fluctuations and ensure grid stability during peak demand. While MILP provides precise solutions, its computational complexity can be prohibitive for large-scale, real-time applications.

Additionally, ref. [9] introduced a two-phase scheduling model for dynamic optimization of EV charging, balancing loads on smart grids while minimizing energy costs and extending battery life. The authors employed stochastic optimization techniques to account for uncertainties in load demand and EV availability. Despite its advantages, this approach may not fully address the dynamic nature of grid conditions and user preferences.

In the realm of demand response strategies, refs. [15,16] developed methods that dynamically adjust charging profiles based on real-time load conditions, considering both grid stability and user satisfaction. While effective, these strategies may not fully account for the complexities of large-scale EV integration and the diverse needs of users.

In the context of smart grid integration, ref. [8] explored the application of reinforcement learning in dynamic charging optimization for EVs, finding that such algorithms can effectively adjust charging patterns according to real-time grid conditions, thereby ensuring grid stability and user preferences. However, the convergence speed and data requirements of reinforcement learning [17,18,19,20] methods remain areas for further research.

Meanwhile, ref. [21] introduced a game-theory-based charging strategy that encourages cooperative behavior among EV users to optimize charging schedules and reduce peak demand. This approach has shown promise in reducing grid congestion and lowering energy costs. Nonetheless, its practical implementation may face challenges related to user cooperation and the dynamic nature of grid conditions.

Chen et al. [22] introduced a method for electric vehicle charging scheduling based on optimal power flow (OPF) and applied the valley-filling strategy to smooth the power demand curve, reduce charging costs, and improve grid stability. Their online algorithm tracks the valley-filling strategy in real time, and its effectiveness has been validated in the IEEE 14-bus system.

Lee et al. [23] proposed the Adaptive Charging Network (ACN) framework, which uses a scheduling algorithm based on convex optimization and model predictive control to address challenges such as infrastructure imbalance and non-ideal battery charging behavior in electric vehicle charging. The framework has been successfully deployed in multiple real-world locations, demonstrating its efficiency in energy management and reducing overload.

### 2.2. Smart Grid and Multi-Objective Optimization

Beyond EV charging scheduling, several studies have explored optimizing smart grid operations using multi-objective optimization techniques. For example, refs. [24,25] proposed an integrated approach to optimal load dispatch and energy storage management in smart grids, using multi-objective optimization to rationally allocate the costs of power generation and energy storage, while ensuring the reliability and stability of the grid. However, these methods may not fully address the complexities of integrating renewable energy sources and EVs into the grid.

Similarly, ref. [26] presented a multi-objective optimization framework for the operation of smart grids, focusing on reducing energy losses, minimizing voltage deviations, and optimizing energy consumption. While comprehensive, this framework may not adequately consider the dynamic and stochastic nature of modern power systems.

Another approach for smart grid optimization was proposed by [27,28,29], which uses evolutionary algorithms to solve the multi-objective optimization issues of voltage regulation and load balancing in a smart distribution system. The methodology considers both grid constraints and operational costs and has been shown to dramatically optimize grid performance. However, evolutionary algorithms can be computationally intensive and may not always converge to the global optimum.

A study by [5,6] introduced an intelligent optimization approach based on genetic algorithms for managing smart grid operations. The authors highlighted the potential of genetic algorithms in solving complex multi-objective optimization problems, especially in the context of large-scale smart grids with multiple stakeholders. Despite their effectiveness, genetic algorithms can suffer from premature convergence and may require fine-tuning of parameters.

Further advancements in optimization techniques and reinforcement learning methods for smart grids are discussed by [10,30,31,32], who proposed a novel hybrid optimization model combining PSO and multi-objective optimization for energy management in smart grids. The model was applied to distributed energy systems, focusing on minimizing energy costs, reducing environmental impact, and enhancing grid stability. However, hybrid models can be complex and may face challenges in real-time implementation.

Additionally, ref. [33] developed a real-time optimization approach for smart grid operation, which integrates forecasting and optimization techniques to improve grid reliability and efficiency under various operational scenarios. While promising, real-time optimization requires accurate forecasting and rapid computational capabilities, which can be challenging to achieve.

Lastly, ref. [34] provided a review of various optimization methods applied to smart grids, discussing the challenges and opportunities in the area of multi-objective optimization. The study argued that multi-objective optimization is essential for solving complex smart grid problems, especially when considering the integration of renewable energy sources and electric vehicles. However, the trade-offs between conflicting objectives remain a significant challenge.

### 2.3. Challenges and Future Directions

While significant progress has been made in the optimization of EV charging schedules, several challenges remain. Many existing methods do not integrate global optimization with real-time feedback in a unified framework. Additionally, few studies consider the voltage and current constraints imposed by real-world distribution networks. The dual-phase optimization strategy proposed in this paper aims to address these gaps by combining PSO for global optimization and Q-learning for real-time adaptation, explicitly incorporating voltage and overload constraints.

## 3. Entropy-Guided Optimization Method

**Figure 1:**
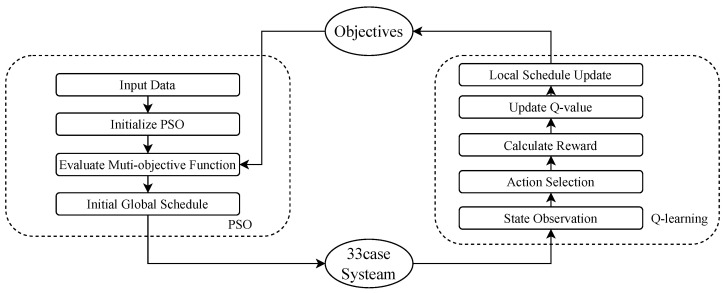
Dual-phase optimization strategy network.

In this section, we describe the methodology for optimizing the voltage quality and mitigating overloading in rural distribution networks, particularly in transitional urban–rural areas, by using a coordinated EV charging strategy. Our model is based on data collection, load modeling, voltage and overload constraints, and particle swarm optimization (PSO). Particle swarm optimization (PSO) gradually converges in the global search, which is similar to the process of gradual entropy reduction, that is, evolution from the initial chaotic multi-solution space (high entropy) to a stable single optimal solution (low entropy) (see Figure 1).

### 3.1. Data Description

**Figure 2:**
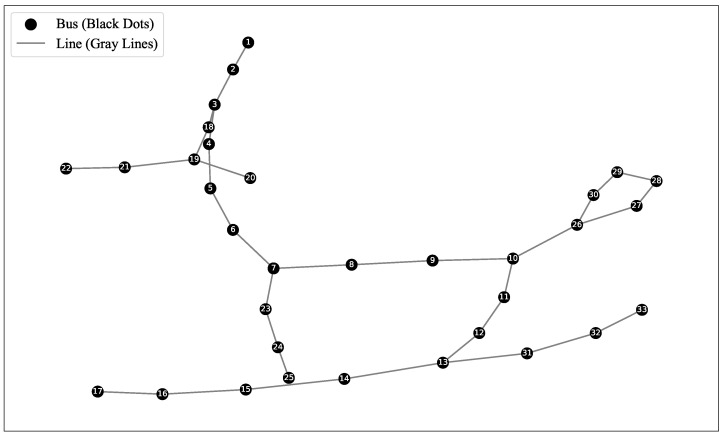
A 33-node distribution system modified based on the load data of the IEEE 39-bus system by removing some low-load nodes.

Smart meters (AMIs) are used to collect hourly consumption data for each node in the network. The data are sampled every hour and the time series length is T (typically 24 h). The base load data $P_{b}\left(t\right)$ represent the typical power consumption of residents, agriculture, and commerce in the distribution network. Historical load data $P_{h}\left(t\right)$ from the past 2 years are used to fit the base load using a weighted moving average method or seasonal decomposition as $P_{b}\left(t\right)=\hat{f}\left(P_{h}\left(t\right)\right)$. The $\hat{f}$ is a regression function (polynomial fitting) to model daily and seasonal variations. The network has N=33 nodes as shown Figure 2, and we collect data for 24 h; the data size is $\left(N\times T\right)\in R^{33\times 24}$ data points [11,12].

The EVs’ charging load is central to our research. We need to model the charging behavior of EVs. Surveys and Charging Station Management Systems (CMSs) are used to gather data on the number of EVs, charging schedules, and charging power. We assume that there are $M\in R^{m}$ EVs in the region, and each EV has a distinct charging demand, characterized by its charging time window $T_{i}$ and charging demand $D_{i}$. The charging power for each EV *i* is calculated based on the charging time window and demand: $P_{\mathrm{EV},i}\left(t\right)=f\left(T_{i},D_{i}\right)$. The $T_{i}$ is the charging time window, typically following a normal distribution or Poisson distribution, and $D_{i}$ is charging demand, which usually due to the battery capacity of the vehicle. The time series data for each EV’s charging load $P_{\mathrm{EV},i}\left(t\right)$ have a length of T hours, with a total data size of $\left(M\times T\right)\in R^{m\times 24}$ data points.

In order to ensure that the EV charging behavior conforms to the capacity of the actual charging facilities and avoid excessive burden on the grid, we need to impose a constraint on the charging power $P_{\mathrm{EV},i}\left(t\right)$ of each EV:

(1) $$\begin{array}{c} 0\leq P_{\mathrm{EV},i}\left(t\right)\leq P_{\mathrm{charger}},\;\forall i,t. \end{array}$$

where the $P_{\mathrm{EV},i}\left(t\right)$ is the charging power of the EV *i* at time *t* and the $P_{\mathrm{charger}}$ is the charging pile’s maximum charging power, depending on the type of charging pile and the load capacity of the grid.

### 3.2. Distribution Network Voltage and Overload Models

The voltage at each node in the distribution network is affected by the charging loads and the base loads. The voltage at node *i* at time *t* is calculated by the following power flow equation:

(2) $$\begin{array}{c} V_{i}\left(t\right)=V_{\mathrm{ref}}-\sum_{j\in N_{i}}\frac{P_{ij}\left(t\right)R_{ij}+Q_{ij}\left(t\right)X_{ij}}{V_{j}\left(t\right)}, \end{array}$$

where $P_{ij}\left(t\right)$ and $Q_{ij}\left(t\right)$ represent the reactive powers between nodes *i* and *j*, which are constant values taken from the network topology. And $R_{ij}$ and $X_{ij}$ are the resistance and reactance of the transmission line between nodes *i* and *j*, which are constant values taken from the network topology. $V_{\mathrm{ref}}$ is the reference voltage, typically set to a standard value such as 220 V or 110 V. $N_{i}$ is the set of all nodes in the network that are electrically connected to node *i*, meaning the nodes that share transmission lines with node *i*.

The voltage at each node must satisfy the following constraints:

(3) $$\begin{array}{c} V_{\min}\leq V_{i}\left(t\right)\leq V_{\max},\;\forall i,t, \end{array}$$

where $V_{\min}$ and $V_{\max}$ are the minimum and maximum allowable voltages for each node.

The goal of this model is to optimize the charging loads of EVs in such a way that the voltage levels across all nodes stay within allowable limits while maintaining efficient energy distribution. The optimization objective is to minimize the voltage deviation at all nodes of the distribution network while ensuring system operation within the permissible voltage range. The voltage deviation at each node is determined by differences among reference voltage $V_{\mathrm{ref}}$ as well as actual voltage at each node $V_{i}\left(t\right)$. The objective function can be formulated as

(4) $$\begin{array}{cc} \min J_{1} & =\sum_{i=1}^{N}{\left(V_{\mathrm{ref}}-V_{i}\left(t\right)\right)}^{2}\\ \mathrm{s.t.}\; & V_{\min}\leq V_{i}\left(t\right)\leq V_{\max},\;\forall i,t, \end{array}$$

where N is all the nodes in the network. Equation (4) aims to minimize the difference between the desired reference voltage and the actual voltage at each node, essentially ensuring voltage stability and reducing fluctuations caused by the charging and base loads. Equation (4) refers to a convex relaxation method based on second-order cone programming (SOCP) proposed by CW Tan et al. to solve the optimal power flow (OPF) objective in resistive networks [35].

To better improve the overall power quality by avoiding voltage-related issues that may harm the electrical equipment and reduce the efficiency of energy distribution and ensure system stability by maintaining voltages within safe operational limits, we use constraints such as Equation (3) to ensure that the voltage at each node stays within the allowable limits, preventing damage to equipment or inefficient power distribution.

Overload in the distribution network is determined by the current flowing through each line. The current $I_{ij}\left(t\right)$ between nodes *i* and *j* is given by

(5) $$\begin{array}{cc} I_{ij}\left(t\right) & =\sqrt{\frac{P_{ij}{\left(t\right)}^{2}+Q_{ij}{\left(t\right)}^{2}}{V_{i}{\left(t\right)}^{2}}},\\ I_{ij}\left(t\right) & \leq I_{\max,ij},\;\forall \left(i,j\right),t, \end{array}$$

where $P_{ij}\left(t\right)$ and $Q_{ij}\left(t\right)$ are the time-varying active and reactive powers. And $V_{i}\left(t\right)$ is the time-dependent voltage at node *i*. The current must satisfy the overload constraint for each transmission line, of which $I_{\max,ij}$ is the maximum current that the transmission line between *i* and *j* can handle.

The overload in the network is a key factor in determining the power distribution. The objective will minimize the overload by penalizing instances where the current exceeds the maximum capacity of the line $I_{\max,ij}$. So, we can obtain the following expression:

(6) $$\begin{array}{cc} \max J_{2} & =\sum_{(i,j)\in E}\max{\left(0,I_{ij}\left(t\right)-I_{\max,ij}\right)}^{2}\\ \mathrm{s.t.}\; & I_{ij}\left(t\right)\leq I_{\max,ij},\;\forall \left(i,j\right),t, \end{array}$$

where E represents all transmission lines’ collection in the network, and $I_{ij}\left(t\right)$ is the current flowing through line i→j.

### 3.3. Optimization Objective and Constraints

This study’s objective is to minimize the voltage deviation, line overload, and maximize user satisfaction with the charging process. In practical applications, user satisfaction is an important factor in the orderly charging strategy of EVs. Charging scheduling of EVs should not only consider the voltage and load of the grid, but also ensure that users can be fully charged in a reasonable time to avoid a long wait for charging. The user satisfaction formula is defined based on the charging completion level, which measures how well the actual charging amount meets the user’s expectations. The formula is derived from user feedback surveys and behavior modeling, where the charging capacity (Egoal) is the target and the actual charging achieved (EEV) is compared. The satisfaction is then defined as the ratio of the actual battery level to the target charging capacity. This method assumes that users are satisfied when their vehicles are fully charged within the specified time window. The rationale behind this approach is that users’ primary satisfaction comes from receiving a sufficient charge, and this can be modeled as a direct comparison of their expectations and the actual outcome. Therefore, user satisfaction is quantified in the following sequential manner:

(7) $$\begin{array}{c} U_{i}=\frac{E_{EV,i}\left(T_{\mathrm{end},i}\right)}{E_{\mathrm{goal},i}}-\beta_{1}\cdot T_{\mathrm{charge},i}-\beta_{2}\cdot T_{\mathrm{wait},i} \end{array}$$

where $E_{EV,i}\left(T_{end,i}\right)$ is the *i*-th battery level of the EV at the end of charging and $E_{goal,i}$ is the *i*-th target charging capacity of the EV. If the charging amount is lower than the target amount, the user fails to obtain the expected charging effect, and the satisfaction is some value between 0 and 1 at this time. The specific satisfaction value $U_{i}$ depends on the degree of charging completion. The user satisfaction formula is extended to account for both charging time and waiting time. The extended formula considers the user’s experience not only in terms of the charging level achieved but also the time it takes to charge and the waiting period before the charging begins. Charging time $T_{\mathrm{charge}}$ is penalized because longer charging times may cause inconvenience, especially for users with higher time sensitivity. Similarly, waiting time $T_{\mathrm{wait}}$ is penalized because users who wait for a long time to start charging are likely to be less satisfied. The weight factors $\beta_{1}$ and $\beta_{2}$ represent the relative importance of charging time and waiting time, respectively, and can be adjusted based on user surveys or operational data. And through the experiment, $\beta_{1}=\beta_{2}=0.01$ obtained the best performance.

So, the overall objective function is as follows in Equations (1), (4), (6) and (7):

(8) $$\begin{array}{ccc} \min J & =\min J_{1}+\max J_{2}+J_{3} & \\ \; & =\alpha_{1}\sum_{i=1}^{N}{\left(V_{\mathrm{ref}}-V_{i}\left(t\right)\right)}^{2}+\alpha_{2}\sum_{(i,j)\in E}\max{\left(0,I_{ij}\left(t\right)-I_{\max,ij}\right)}^{2}-\alpha_{3}\sum_{i=1}^{M}U_{i},\\ \mathrm{s.t.}\; & \left\{\begin{array}{c} 0\leq P_{\mathrm{EV},i}\left(t\right)\leq P_{\mathrm{charger}},\;\forall i,t.\\ V_{\min}\leq V_{i}\left(t\right)\leq V_{\max},\;\forall i,t,\\ I_{ij}\left(t\right)\leq I_{\max,ij},\;\forall \left(i,j\right),t, \end{array}\right. \end{array}$$

where $\alpha_{1},\alpha_{2},\alpha_{3}$ are weighting factors that balance the voltage, overload, and user satisfaction terms. The optimization problem is subject to several constraints that ensure both system stability and user satisfaction. The first constraint ensures that the charging power $P_{\mathrm{EV},i}\left(t\right)$ for every EV at time *t* is non-negative and does not exceed the maximum allowable charging power of the charging pile, $P_{\mathrm{charger}}$, as expressed in Equation (1). This ensures that the charging process is feasible without overloading the charging infrastructure. The second set of constraints ensures voltage stability across the network. Specifically, the voltage at each node $V_{i}\left(t\right)$ is required to stay within a specified range, $V_{\min}\leq V_{i}\left(t\right)\leq V_{\max}$, to avoid overvoltage or undervoltage conditions that could harm electrical equipment or cause inefficient power distribution. Finally, the third set of constraints limits the current flow between any two nodes $I_{ij}\left(t\right)$ to be below the maximum allowable current capacity $I_{\max,ij}$, as described in Equation (6). This ensures that the power lines do not exceed their maximum rated capacity, preventing overloads that could compromise the safety and stability of the distribution network. Together, these constraints guarantee that the optimization process will not only minimize voltage deviation and line overload but also maintain the safety and reliability of the electrical network while achieving optimal user satisfaction.

### 3.4. Dual-Phase Entropy Optimization Strategy

A two-phase optimization approach is proposed to optimize the EV charging schedule in rural distribution networks, where the goal is to achieve a balance between voltage stability, load management, and user satisfaction. By iteratively refining the charging strategy, this dual-phase system improves the orderliness of power distribution and reduces fluctuations in grid conditions. The first phase utilizes PSO to achieve a globally optimal charging schedule by minimizing voltage deviation and line overload, while maximizing user satisfaction. The second phase uses Q-learning technology to dynamically adjust the charging schedule in accordance with continuous feedback from the distribution network in real time. Q-learning dynamically adjusts the charging strategy through reinforcement learning, which also optimizes the uncertainty of the strategy under real-time system feedback and gradually reduces the entropy value.

PSO is a population-based optimization algorithm inspired by the social behavior of birds and fish. PSO is well suited for multi-objective sequential optimization issues and is therefore ideal for the initial optimization of EV charging schedules in this study. In the first phase, PSO optimizes the initial charging plan by minimizing voltage deviations and line overloads and maximizing user satisfaction, considering the distribution network’s physical constraints.

PSO is operated by iteratively adjusting a population of candidate solutions in the search space. This iterative refinement helps the system converge toward a more stable and balanced charging schedule, reducing the risk of extreme voltage deviations or overloads. Every particle stands for a potential charging schedule, and its position and velocity define the schedule and its adjustments, respectively. The algorithm tracks the best solution found by each particle as its personal best and the best solution found by the entire swarm as the global best.

The position and velocity of each particle are updated at each iteration as follows:

(9) $$\begin{array}{cc} v_{i}\left(t+1\right) & =w\cdot v_{i}\left(t\right)+c_{1}r_{1}\left(p_{\mathrm{best},i}-x_{i}\left(t\right)\right)+c_{2}r_{2}\left(g_{\mathrm{best}}-x_{i}\left(t\right)\right),\\ x_{i}\left(t+1\right) & =x_{i}\left(t\right)+v_{i}\left(t+1\right), \end{array}$$

where $v_{i}\left(t\right)$ and $x_{i}\left(t\right)$ represent the velocity and position of particle *i* at iteration *t*, respectively. *w* is the inertia weight, $c_{1}$ and $c_{2}$ are cognitive and social learning factors, and $r_{1},r_{2}$ are random numbers sampled from [0,1]. The global best $g_{\mathrm{best}}$ as well as personal best $p_{\mathrm{best},i}$ guide a swarm toward optimal solutions.

PSO algorithms for EV charging schedule global optimization are detailed in Algorithm 1.
**Algorithm 1** Particle swarm optimization (PSO) for EV charging scheduling  1:**Input:** Swarm size $N_{\mathrm{swarm}}$, maximum iterations $T_{\max}$, inertia weight *w*, cognitive factor $c_{1}$, social factor $c_{2}$  2:**Initialize:** Randomly initialize particles’ positions $x_{i}\left(0\right)$ and velocities $v_{i}\left(0\right)$  3:Set personal best positions $p_{\mathrm{best},i}$ and global best $g_{\mathrm{best}}$  4:**for** iteration t=1 to $T_{\max}$ **do**  5:    **for** each particle *i* in swarm **do**  6:        Update velocity:$$v_{i}\left(t+1\right)=w\cdot v_{i}\left(t\right)+c_{1}r_{1}\left(p_{\mathrm{best},i}-x_{i}\left(t\right)\right)+c_{2}r_{2}\left(g_{\mathrm{best}}-x_{i}\left(t\right)\right)$$  7:        Update position:$$x_{i}\left(t+1\right)=x_{i}\left(t\right)+v_{i}\left(t+1\right)$$  8:        Evaluate fitness based on objective function  9:        **if** fitness improves personal best **then**10:           Update $p_{\mathrm{best},i}$11:        **end if**12:        **if** fitness improves global best **then**13:           Update $g_{\mathrm{best}}$14:        **end if**15:    **end for**16:**end for**17:**Output:** Optimal charging schedule $g_{\mathrm{best}}$

Key parameters such as the swarm size $N_{\mathrm{swarm}}$, inertia weight *w*, and learning factors $c_{1},c_{2}$ are tuned to ensure convergence. The fitness of every particle is assessed by the multi-objective function defined in Equation (1). By iteratively updating positions and velocities, the algorithm converges toward an optimal charging schedule that balances voltage deviation, overload mitigation, and user satisfaction.

- Q-learning for Real-Time Adjustment

Once the initial charging schedule has been optimized using PSO, the second phase utilizes Q-learning to dynamically adjust the charging schedules in response to real-time feedback from the grid. This adaptation is necessary due to the dynamic nature of the distribution network, where load conditions and the number of active EVs can change over time. This adaptation allows the system to handle dynamic changes in grid conditions, such as varying load levels and user charging demands, while maintaining system stability. Q-learning, a model-free RL algorithm, is particularly well-suited for this task because it does not require a model of the environment. Instead, it learns an optimal policy by interacting with the environment and adjusting its actions based on the observed rewards. The agent learns from its experiences to optimize the long-term reward, which, in this case, is the combination of stable voltage levels, minimized overload, and maximum user satisfaction.

The Q-learning algorithm operates by updating the Q-value function Q(s,a) iteratively, where *s* is the state and *a* is the action taken in that state. The update rule is

(10) $$\begin{array}{cc} Q(s_{t},a_{t}) & \leftarrow Q\left(s_{t},a_{t}\right)+\alpha \left[r_{t}+\gamma \max_{a}Q\left(s_{t+1},a\right)-Q\left(s_{t},a_{t}\right)\right], \end{array}$$

where α is the learning rate, γ is the discount factor, $r_{t}$ is the immediate reward, and $\max_{a}Q\left(s_{t+1},a\right)$ is the maximum Q-value for the next state.

The Q-learning algorithm for real-time charging schedule adjustment is presented in Algorithm 2.
**Algorithm 2** Q-learning for real-time charging scheduling  1:**Input:** Learning rate α, discount factor γ, exploration rate ϵ, maximum episodes $E_{\max}$  2:**Initialize:** Q-table Q(s,a) with zeros for all state–action pairs  3:**for** episode e=1 to $E_{\max}$ **do**  4:    Initialize state $s_{0}$  5:    **for** each time step *t* **do**  6:        Select action $a_{t}$ using ϵ-greedy policy  7:        Execute action $a_{t}$, observe reward $r_{t}$ and next state $s_{t+1}$  8:        Update Q-value:$$Q\left(s_{t},a_{t}\right)\leftarrow Q\left(s_{t},a_{t}\right)+\alpha \left[r_{t}+\gamma \max_{a}Q\left(s_{t+1},a\right)-Q\left(s_{t},a_{t}\right)\right]$$  9:        **if** termination condition met **then**10:           Break11:        **end if**12:    **end for**13:**end for**14:**Output:** Optimal policy $\pi^{*}\left(s\right)=\arg\max_{a}Q\left(s,a\right)$

Where state $s_{t}$ includes voltage levels, grid load, and active EV connections. Action $a_{t}$ represents the charging power adjustment for each EV. The reward $r_{t}$ encourages voltage stability as defined in Equation (4), avoids overload as specified in Equation (6), and improves user satisfaction according to Equation (7).

The proposed two-phase strategy combines PSO and Q-learning to achieve both global and real-time optimization of EV charging schedules. PSO provides an efficient initial solution, while Q-learning dynamically adapts to grid conditions, ensuring stability, efficiency, and user satisfaction.

The transition from PSO to Q-learning is crucial for adapting the charging schedule based on the dynamic nature of the grid. While PSO provides an initial globally optimal charging schedule, it does not account for real-time fluctuations in voltage and load, which are critical in a distribution network with EVs. By continuously adjusting charging power in response to grid feedback, the system reduces the likelihood of voltage instability and overload, leading to a more predictable and efficient power distribution process. This coordinated approach minimizes unnecessary fluctuations and contributes to a more reliable and orderly grid operation.

PSOs are able to minimize voltage deviations and line overloads across the grid, as well as maximize user satisfaction, by optimizing charging schedules by taking global factors into account. However, the state of the grid can change over time due to changes in demand, grid faults, or changes in the number of EVs, requiring Q-learning to dynamically adjust the charging plan.

In the second phase of the optimization strategy, Q-learning refines the charging schedule in real time by continuously adapting to the feedback from the network. Through the iterative process of Q-learning, the agent learns to adapt each EV’s charging power to voltage variations, overload conditions, and user requirements. The optimal strategy, learned from real-time interactions with the environment, allows for dynamic balancing of the network, maintaining stability and maximizing user satisfaction under varying grid conditions.

In this part, we present an integrated approach. We use a two-stage optimization strategy combining particle swarm optimization (PSO) and Q-learning to achieve global optimization and real-time adaptive control of the charging process. It is able to optimize the EV charging schedule in rural distribution networks in urban–rural transition areas.

During the first stage, in order to minimize voltage deviation, reduce line overload, and maximize user satisfaction, we use the PSO algorithm to achieve global optimization for EV charging schedules by iteratively adjusting the EV charging schedule across the network to provide the best initial solution. The solution is formulated as a multi-objective optimization problem with constraints on voltage levels and line currents to ensure that the charging process does not lead to grid instability. In the second phase, we apply a reinforcement learning algorithm, Q-learning, which adjusts the charging plan in real time based on dynamic feedback from the grid. It adjusts the charging power of each EV based on real-time grid conditions such as voltage fluctuations and load changes, ensuring a continuous balance between grid stability and user charging demand.

The final output is a dual-phase optimized charging strategy that dynamically balances grid stability and user satisfaction. This strategy takes the form of a detailed charging schedule for each EV, with specific charging times and power levels. The charging schedule for each EV has a size $M\times T\in R^{m\times 24}$, which covers all charging stations and EVs in the network, ensuring that the grid operates within safe voltage and current limits while optimizing for user charging satisfaction.

## 4. Experiment

**Figure 3:**
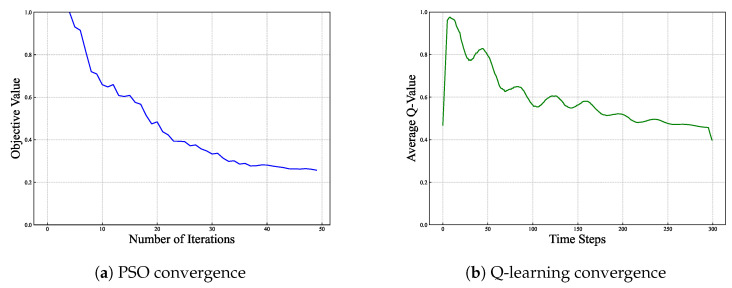
Algorithm convergence analysis. Voltage deviation reduction (**a**) and entropy dynamics (**b**). The entropy curve in (**b**) aligns with voltage stabilization, confirming the theoretical framework.

The specific parameter values used in the experiments are shown in Table 1. In the baseline scenario, the system includes 10 electric vehicles distributed across nodes 5, 8, 10, 15, 20, 23, and 25. In the high-load scenario, the system contains 15 electric vehicles, with an additional 5 vehicles concentrated around the main line at nodes 4, 7, 11, 13, and 18. The charging power for the electric vehicles is set at 7.2 kW. We evaluated the proposed two-stage optimization algorithm for its performance and efficiency in various scenarios. We first analyzed the convergence properties of the particle swarm optimization (PSO) and Q-learning algorithms, which are the core components of the proposed framework, as shown in Figure 3.

Figure 3a describes the decrease of the objective function value with the number of iterations. As shown, the PSO algorithm achieves convergence after approximately 50 iterations, with the objective function value stabilizing around 0.3. Figure 3b displays the Q-learning algorithm’s convergence, where cumulative rewards grow as the number of episodes grows. The results demonstrate that Q-learning requires approximately 300 episodes to achieve a stable policy, highlighting its capability to adapt to dynamic scenarios through iterative learning.

To better simulate normal charging load demands, we tested the model under both the baseline scenario and the high-load scenario. The baseline scenario simulates the daily operational state of a 33-node distribution network under typical working conditions. Load and electric vehicle charging demands vary over a 24 h period. The high-load scenario models the grid’s performance under extreme conditions during evening peak hours (17:00–21:00). This scenario assumes simultaneous charging of 15 electric vehicles (EVs), imposing significant stress on the system. The objective is to evaluate the proposed optimization algorithm’s ability to maintain voltage stability, reduce line load rates, and improve user satisfaction under both high-demand and extreme high-demand conditions.

**Figure 4:**
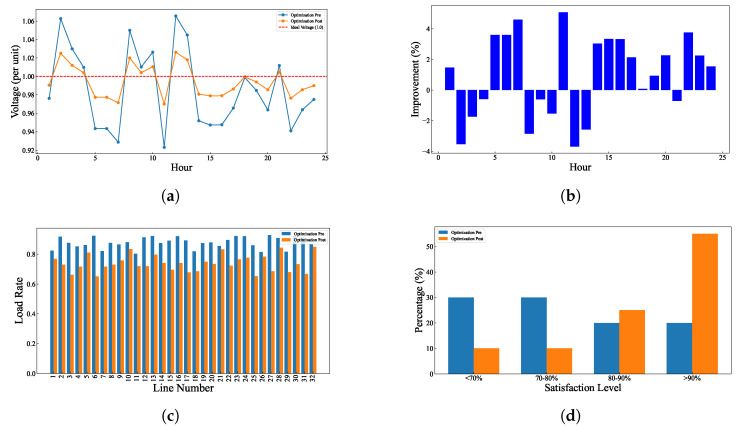
Comparison of line load, user satisfaction, voltage, and voltage improvement in baseline scenario. (**a**) Voltage comparison at each node in baseline scenario. (**b**) Voltage improvement at each node in baseline scenario. (**c**) Line load comparison in baseline scenario. (**d**) User satisfaction comparison in baseline scenario.

The test results of the model under the baseline scenario are shown in Figure 4. Figure 4a shows the voltage comparison before and after optimization. Before optimization, voltage fluctuations are large, sometimes exceeding or falling below the ideal 1.0 unit. After optimization, the voltage curve becomes much smoother, staying close to the 1.0 unit target throughout the day. This indicates that the optimization measures effectively regulate the voltage within the desired range. Figure 4b shows the percentage improvement in voltage achieved through optimization. Most of the time, the voltage improvement is around 2–4%, with improvements exceeding 5% during midday. This indicates that the optimization significantly enhances voltage performance. Figure 4c compares the line load rates before and after optimization. After optimization, load distribution becomes more uniform, and the peak load on the busiest lines is reduced. This helps improve the efficiency and stability of the entire power system. Figure 4d shows the impact of optimization on user satisfaction. Before optimization, about 30% of users have a satisfaction below 70%, but this proportion drops to less than 10% after optimization. Meanwhile, the proportion of highly satisfied users (above 90%) increases from about 15% to over 40%. This indicates that the optimization directly improves user experience and satisfaction.

**Figure 5:**
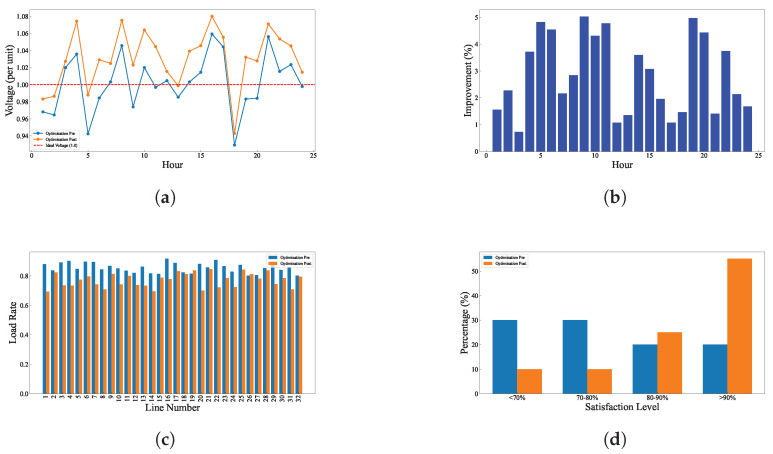
Comparison of line load, user satisfaction, voltage, and voltage improvement in high-load scenario. (**a**) Voltage comparison at each node in high-load scenario. (**b**) Voltage improvement at each node in high-load scenario. (**c**) Line load comparison in high-load scenario. (**d**) User satisfaction comparison in high-load scenario.

The test results of the model under the high-load scenario are shown in Figure 5. As shown in Figure 5a, the optimized voltage curve significantly stabilizes, with a noticeable reduction in fluctuation amplitude. Additionally, as shown in Figure 5b, the voltage improvement reaches around 4% during midday. This indicates that even under high-load conditions, optimization measures can still play a role in enhancing voltage stability. In terms of line load, as shown in Figure 5c, the load distribution among lines improves after optimization, but it still remains relatively concentrated on a few lines. This suggests that the pressure from high load limits the effectiveness of optimization measures in achieving balanced line load distribution. However, compared to the pre-optimization situation, there has been a significant improvement. From the perspective of user satisfaction, as shown in Figure 5a, the proportion of users with lower satisfaction decreases after optimization. This indicates that even under high-load conditions, the improvement in system performance can enhance user experience to some extent.

Additionally, we conducted comparative trials. Table 2 compares three scheduling methods. The two-stage approach demonstrates superior performance with a voltage deviation of 1.9%, compared to 5.8% for FCFS and 3.2% for standalone PSO. The two-stage approach reduces the voltage deviation from 5.8% to 1.9%. This improvement verifies the effectiveness of the entropy minimization framework: a decrease in voltage deviation directly corresponds to a decrease in system disorder. Different from the strategy of Chen et al. [11] to reduce the entropy value through the integration of renewable energy, this work proves, for the first time, the applicability of entropy theory in dynamic EV scheduling, which provides a new stability guarantee mechanism for rural power grids. Maximum load rates decreased from 95% with FCFS to 82% with the two-stage method, while user satisfaction increased from 75% to 88%. Although the two-stage method requires longer computation time, its overall performance improvements justify this trade-off.

## 5. Conclusions

This study proposes a dual-phase optimization strategy that successfully addresses the key challenges of electric vehicle charging scheduling in rural distribution networks. By explicitly minimizing system entropy as a core concept, we achieved significant improvements in grid stability and user satisfaction. Experimental results on a 33-node distribution system demonstrate that our dual-phase PSO-Q learning method outperforms both traditional methods and standalone PSO: voltage deviation decreased from 5.8% for FCFS and 3.2% for standalone PSO to 1.9%, maximum load factor reduced from 95% to 82%, and average user satisfaction improved from 75% to 88%. These improvements directly verify the effectiveness of the entropy minimization framework, showing a direct correlation between reduced system disorder and improved voltage stability.

The Q-learning-enhanced real-time adjustment mechanism enables the system to dynamically respond to changing grid conditions, performing exceptionally well in high-load scenarios and demonstrating robustness in variable environments. Although the computation time of 66 min is longer than standalone PSO at 34 min, this trade-off is reasonable and practical considering the magnitude of performance improvement and the time scale of charging scheduling in practical applications.

This research not only provides a practical solution for EV charging scheduling but, more importantly, establishes a grid management framework based on entropy theory, offering a viable and robust pathway for large-scale integration of EVs into rural power systems. Future work will explore applications of this method in larger-scale systems and the possibility of incorporating renewable energy generation and storage systems into the optimization framework.

## Figures and Tables

**Table 1:** Experiment parameter settings.

Algorithm	Parameter	Value
PSO	Number of particles	50
	Maximum iterations	100
	Inertia weight (*w*)	0.7
	Cognitive factor ($c_{1}$)	1.5
	Social factor ($c_{2}$)	1.5
Q-learning	Learning rate (α)	0.1
	Discount factor (γ)	0.89
	Exploration rate (ϵ)	0.0001
	Maximum episodes	300
Multi-objective weights	Voltage deviation ($\alpha_{1}$)	0.51
	Line overload ($\alpha_{2}$)	0.36
	User satisfaction ($\alpha_{3}$)	0.29

**Table 2:** Performance Comparison of scheduling methods.

Metric	Traditional FCFS	Standalone PSO	Two-Stages
Voltage Deviation (%)	5.8	3.2	1.9
Max Load Rate (%)	95	88	82
Avg. Satisfaction (%)	75	82	88
Computation Time (min)	-	34	66

## Data Availability

The data presented in this study are available on request from the corresponding author.

## Nomenclature

**Symbol**	**Description**
$P_{b}\left(t\right)$	Base load data at time *t*
$P_{h}\left(t\right)$	Historical load data at time *t*
$\hat{f}$	Regression function for modeling load variations
*N*	Number of nodes in the distribution network (33 nodes in this study)
*T*	Time series length (24 h)
*M*	Number of electric vehicles in the region
$T_{i}$	Charging time window for EV *i*
$D_{i}$	Charging demand for EV *i*
$P_{EV,i}\left(t\right)$	Charging power of EV *i* at time *t*
$P_{charger}$	Maximum charging power of charging pile
$V_{i}\left(t\right)$	Voltage at node *i* at time *t*
$V_{ref}$	Reference voltage (standard value, e.g., 220 V or 110 V)
$P_{ij}\left(t\right)$	Active power between nodes *i* and *j* at time *t*
$Q_{ij}\left(t\right)$	Reactive power between nodes *i* and *j* at time *t*
$R_{ij}$	Resistance of transmission line between nodes *i* and *j*
$X_{ij}$	Reactance of transmission line between nodes *i* and *j*
$N_{i}$	Set of nodes electrically connected to node *i*
$V_{min}$	Minimum allowable voltage
$V_{max}$	Maximum allowable voltage
$J_{1}$	Objective function for voltage deviation minimization
$I_{ij}\left(t\right)$	Current between nodes *i* and *j* at time *t*
$I_{max,ij}$	Maximum current capacity for transmission line between nodes *i* and *j*
E	Collection of all transmission lines in the network
$J_{2}$	Objective function for overload minimization
$U_{i}$	User satisfaction for EV *i*
$E_{EV,i}\left(T_{end,i}\right)$	Battery level of EV *i* at the end of charging
$E_{goal,i}$	Target charging capacity of EV *i*
$T_{charge,i}$	Charging time for EV *i*
$T_{wait,i}$	Waiting time for EV *i* before charging begins
$\beta_{1},\beta_{2}$	Weight factors for charging time and waiting time in user satisfaction
*J*	Overall objective function
$J_{3}$	User satisfaction objective function
$\alpha_{1},\alpha_{2},\alpha_{3}$	Weighting factors for voltage, overload, and user satisfaction terms
$v_{i}\left(t\right)$	Velocity of particle *i* at iteration *t* in PSO algorithm
$x_{i}\left(t\right)$	Position of particle *i* at iteration *t* in PSO algorithm
*w*	Inertia weight in PSO algorithm
$c_{1},c_{2}$	Cognitive and social learning factors in PSO algorithm
$r_{1},r_{2}$	Random numbers sampled from [0, 1] in PSO algorithm
$p_{best,i}$	Personal best position for particle *i* in PSO algorithm
$g_{best}$	Global best position in PSO algorithm
$N_{swarm}$	Swarm size in PSO algorithm
$T_{max}$	Maximum iterations in PSO algorithm
Q(s,a)	Q-value function for state *s* and action *a* in Q-learning
α	Learning rate in Q-learning algorithm
γ	Discount factor in Q-learning algorithm
$r_{t}$	Immediate reward at time step *t* in Q-learning
ϵ	Exploration rate in Q-learning algorithm
$E_{max}$	Maximum episodes in Q-learning algorithm
$\pi^{*}\left(s\right)$	Optimal policy derived from Q-learning
